# Effects of Cerium and Titanium Oxide Nanoparticles in Soil on the Nutrient Composition of Barley (*Hordeum vulgare* L.) Kernels

**DOI:** 10.3390/ijerph13060577

**Published:** 2016-06-09

**Authors:** Filip Pošćić, Alessandro Mattiello, Guido Fellet, Fabiano Miceli, Luca Marchiol

**Affiliations:** Department of Agriculture, Food, Environment and Animal Sciences, University of Udine, Via Delle Scienze 206, 33100 Udine, Italy; alessandro.mattiello@uniud.it (A.M.); guido.fellet@uniud.it (G.F.); fabiano.miceli@uniud.it (F.M.); marchiol@uniud.it (L.M.)

**Keywords:** cerium oxide nanoparticles, titanium oxide nanoparticles, barley, amino acids, amylose, crude protein, macronutrients, micronutrients

## Abstract

The implications of metal nanoparticles (MeNPs) are still unknown for many food crops. The purpose of this study was to evaluate the effects of cerium oxide (*n*CeO_2_) and titanium oxide (*n*TiO_2_) nanoparticles in soil at 0, 500 and 1000 mg·kg^−1^ on the nutritional parameters of barley (*Hordeum vulgare* L.) kernels. Mineral nutrients, amylose, β-glucans, amino acid and crude protein (CP) concentrations were measured in kernels. Whole flour samples were analyzed by ICP-AES/MS, HPLC and Elemental CHNS Analyzer. Results showed that Ce and Ti accumulation under MeNPs treatments did not differ from the control treatment. However, *n*CeO_2_ and *n*TiO_2_ had an impact on composition and nutritional quality of barley kernels in contrasting ways. Both MeNPs left β-glucans unaffected but reduced amylose content by approximately 21%. Most amino acids and CP increased. Among amino acids, lysine followed by proline saw the largest increase (51% and 37%, respectively). Potassium and S were both negatively impacted by MeNPs, while B was only affected by 500 mg *n*CeO_2_·kg^−1^. On the contrary Zn and Mn concentrations were improved by 500 mg *n*TiO_2_·kg^−1^, and Ca by both *n*TiO_2_ treatments. Generally, our findings demonstrated that kernels are negatively affected by *n*CeO_2_ while *n*TiO_2_ can potentially have beneficial effects. However, both MeNPs have the potential to negatively impact malt and feed production.

## 1. Introduction

Nanotechnology is expected to be in widespread use by 2020, promoting a market of three trillion dollars’ worth of nanotechnology-based products with six million workers [1]. The applications of nanotechnologies in medicine, consumer goods, heavy industry, information and communication technologies, electronic devices, and environmentally friendly energy systems are developing at a much faster pace than our knowledge of their impact [2]. Several questions have been raised about the fate of nanomaterials (defined as materials with at least one dimension between 1 and 100 nm) [3] used in the agro-environment and those resulting from uncontrolled or accidental flows such as metal nanoparticles (MeNPs). For this reason, the Food and Agriculture Organization of the United Nations (FAO), as well as many countries adhering to the Organization for Economic Co-operation and Development (OECD), recognized the need for early research to fill the existing gaps in knowledge on the toxicity of nanoparticles, their bioaccumulation, oral exposure and risks of ingestion by target organisms, which are the key factors needed for risk assessment of nanomaterials [4,5].

Human diets obtain minerals primarily from grains. However, uptake of nutrients by plant roots is affected by abiotic and biotic stressors and so is the mineral storage in plant organs [6]. It is therefore appropriate to examine whether MeNPs in soil are able to influence mineral accumulation in the grains of food crops. Vascular plants and especially crops are of concern as they could be exposed to risks from MeNPs bioaccumulation and their subsequent entry into the food chain [7,8]. The effects of MeNPs on plants will depend, among other things, on the soil type and plant species [9], and indeed contrasting results are reported among dicot and monocot plants [10,11,12,13,14,15,16]. It is therefore crucial to investigate the impact of MeNPs on crops.

Cerium oxide (*n*CeO_2_) and titanium oxide (*n*TiO_2_) nanoparticles are both included in the list of engineered nanomaterials with priority for immediate testing [17]. A number of papers report data collected in the course of short experiments exposing plants to *n*CeO_2_ or *n*TiO_2_ conducted on seedlings in petri-dishes [18,19,20,21], in hydroponic solutions or perlite-containing pots [11,22,23,24,25,26,27,28], and in nutrient medium in agar [29,30]. Other scientists worked on experiments in pots with plants being sprayed with *n*TiO_2_ on leaves [31,32,33,34,35]. Lastly, Wang *et al.* [36] and the Gardea-Torresdey group published research on the effects of *n*CeO_2_ and *n*TiO_2_ on the yields of different plant species in soil experiments [11,15,16].

This paper reports the observations of barley (*Horedum vulgare* L.) kernels produced by plants grown in *n*CeO_2_ or *n*TiO_2_ amended soil. Barley is among the world’s most important crops, being the fourth cereal after maize, rice and wheat for global grain production in 2013 [37]. Due to its adaptation, N uptake and utilization efficiency (N_UtE_), barley is cultivated on more than 49 million hectares [37,38]. It has relevant economic importance for animal feed, malting and brewing and is an important food in some parts of the world [37].

The main aims of our experiment were to: (i) evaluate the bioaccumulation of Ce and Ti in kernels; (ii) verify the mineral nutrients content in kernels compared to that in control plants; and (iii) monitor the changes, if any, in seed quality parameters.

## 2. Materials and Methods

### 2.1. Soil Characterization

The soil used in our pot experiment was collected from the top 40 cm of an agricultural field at the University of Udine, Italy (46°04′53″ N, 13°12′34″ E). The soil was air dried and sieved through a 2-mm sieve. For the soil characterization, samples (5 replicates) were oven-dried at 40 °C for 48 h. The samples were then analyzed according to Pansu and Gautheyrou [39] for particle size distribution (Bouyoucos hydrometer method), pH (potentiometric measurements in a 1 to 2.5 of soil and Milli-Q^®^ water suspension), electrical conductivity (EC, conductometric measurements in a 1 to 5 of soil and Milli-Q^®^ water suspension), cation exchange capacity (CEC), available P (sodium bicarbonate extractable P at pH 8.5, Olsen method) and equivalent carbonate (calcimeter method). Total organic carbon (TOC) and total nitrogen (TN) contents were determined through an Elemental CHNS Analyzer (Vario Micro Cube, Elementar Analysensysteme GmbH, Hanau, Germany) using up to 10 mg of finely ground soil. Carbonates from the soil were previously removed by adding drops of hydrochloric acid (18%). The soil was classified as sandy clay loam and its characteristics are reported in Table 1.

### 2.2. Nanoparticles Characteristics

Cerium(IV) oxide (*n*CeO_2_) and titanium(IV) oxide anatase (*n*TiO_2_) nanoparticles were purchased from Sigma-Aldrich (Milwaukee, WI, USA) (ID product 544841 and 637254, respectively), which described them as having mean diameters of <25 nm (BET). Previous characterization showed that *n*CeO_2_ and *n*TiO_2_ are of different shapes, mainly rhombus and disks, with an average size ±SE of 22.7 ± 1.3 nm and 24.1 ± 0.7 nm (TEM). The specific surface area of *n*CeO_2_ and *n*TiO_2_ resulted as being 46.1 m^2^·g^−1^ and 61.6 m^2^·g^−1^ (BET), respectively. The size distribution of the nanoparticle powders by AFM and in aqueous metrics by DCS, zeta potential and size in aqueous metrics by DLS have been reported previously [40].

### 2.3. Plant Growth and Yield Parameters

Nanoparticles were added to the soil before sowing [40]. Four mixtures of soil were prepared with *n*CeO_2_ or *n*TiO_2_ at 500 and 1000 mg MeNPs·kg^−1^. The control treatment received no MeNPs. Five polyethylene pots (4 L each) were prepared per treatment (for a total of 25 pots). The range of concentrations (0, 500, and 1000 mg MeNPs·kg^−1^) was chosen considering that nanomaterial content in soil is expected to grow in the future due to anthropic activities [41]. Similar concentrations are also reported in numerous articles dealing with nanoparticles interaction with higher plants [42].

A greenhouse experiment was initiated at the experimental farm of the University of Udine (Italy) on 9 April 2014. Seeds of a two-row spring barley (*Hordeum vulgare* L., var. Tunika) obtained from S.I.S. S.p.A. (San Lazzaro di Savena, Bologna, Italy) were sown in pots [40]. More details regarding the experimental design, soil, and greenhouse conditions were reported in a previous paper [40].

All seeds germinated and pots were watered to maintain the soil at 60% of water holding capacity (WHC). During growth pots were individually weighed and watered on a weekly basis to compensate for evapotranspiration. At Zadoks growth stage 92 (Ripening, kernel is hard and can no longer be dented by thumb-nail) [43], kernels were harvested, counted and weighed for 100-kernels weight and grain yield estimation. Flag leaf area was measured using a LI-3100C Area Meter (Li-Cor Corporation, Lincoln, NE, USA). Kernels from the main shoot were then separated for the analyses described below. A subsample of kernels was oven-dried at 105 °C for 48 h for ICP-AES analysis while for the other analyses kernels were oven-dried at 60 °C for 48 h and ground to fine powder.

### 2.4. Amylose and β-Glucans Concentrations Analysis

Amylose and β-glucans concentrations were determined using the enzyme-specific amylose/amylopectin kit and the mixed linkage β-glucans assay kit, both from Megazyme (Megazyme International Ltd., Bray, Ireland).

### 2.5. Amino Acid and Crude Protein Analysis

Acid hydrolysis with HCl at 110 °C for 22–24 h was used for total amino acids except for sulfur amino acids and tryptophan. For sulfur amino acids, performic acid oxidation for 16 h followed by acid hydrolysis with HCl was used. For tryptophan, alkaline hydrolysis with sodium hydroxide was performed at 100 °C for 4 h. After extraction, samples were derivatized at 55 °C for 10 min with 20 μL of AccQ-Fluor reagent (6-aminoquinolyl-*N*-hydroxysuccinimidyl carbamate) and injected in HPLC [44]. All reagents were of HPLC grade. Amino acids analysis was performed using a LC 200 Perkin Elmer pump fitted with an ISS-100 auto sampler (20 µL loop) and a fluorimetric detector (Perkin Elmer, Norwalk, CT, USA), λ excitation of 250 nm and λ emission of 395 nm. Separation was achieved by using one AccQ-Tag amino acid analysis column (Waters Corporation, Milford, MA, USA) and one Waters pre-column filter. The column was thermostatted at 37 °C and the flow rate was 800 μL·min^−1^ [45]. Mobile phase A consisted of acetate-phosphate aqueous buffer, mobile phase B was acetonitrile 100% and eluent phase C was Milli-Q^®^ water. l-α-amino-*n*-butyric acid was used as internal standard. For the analysis of cysteine, methionine and tryptophan it was necessary on some occasions to mix two or three samples to obtain sufficient material for analysis.

Total N content was determined through an Elemental CHNS Analyzer (Vario Micro Cube, Elementar Analysensysteme GmbH, Hanau, Germany) using up to 2.5 mg of finely ground samples. Lastly, crude protein (CP) was estimated by multiplying the nitrogen content by 5.45 [46].

### 2.6. Element Concentrations Analysis

About 300 mg of material were digested in 10 mL of a 1 to 4 (*v*/*v*) mixture of 37% (*v*/*v*) HCl (99.999% trace metals basis, Sigma-Aldrich) and 67% (*v*/*v*) HNO_3_ (RS superpure for trace analysis, Carlo Erba Reagents, Cornaredo, Italy) in Teflon cylinders for 10 min at 175 °C in a microwave oven (CEM, MARS Xpress, Matthews, NC, USA). After mineralization plant extracts were filtered (0.45 μm PTFE) and diluted. Total B, Ca, Cu, Fe, K, Mg, Mn, Na, Ni, P, and Zn contents were determined by an ICP-AES (Vista MPX, Varian Inc., Palo Alto, CA, USA) with an internal standard solution of Y. Total Ce and Ti contents were determined by an ICP-MS (Aurora M90, Bruker, Bremen, Germany) with an internal standard solution of ^72^Ge and ^89^Y. The accuracy of the analytical procedure adopted for ICP-AES analysis was checked by running standard solutions every 20 samples. Quality control for both ICP-AES and ICP-MS was performed using reagent blank samples, and triplicate readings for each sample. Certified standard reference material (tomato leaves 1573a from the National Institute of Standards and Technology, Gaithersburg, MD, USA) was treated as the samples (*n* = 3). The recovery of the elements in the standard material was on average 97% of the certified values with an RSD average of 1.3%. Method detection limits in ICP-AES were: 160 μg·L^−1^, 56 μg·L^−1^, 4 μg·L^−1^, 10 μg·L^−1^, 21 μg·L^−1^, 77 μg·L^−1^, 110 μg·L^−1^, 3 μg·L^−1^, 54 μg·L^−1^, 8 μg·L^−1^, 31 μg·L^−1^, 5 μg·L^−1^ and 6 μg·L^−1^ for Ca, Ce, Cu, B, Fe, K, Mg, Mn, Na, Ni, P, Ti and Zn respectively. Method detection limit in ICP-MS was 1 μg·L^−1^ for both Ce and Ti.

Total S content was determined through an Elemental CHNS Analyzer (Vario Micro Cube, Elementar Analysensysteme GmbH, Hanau, Germany) using up to 2.5 mg of finely ground samples.

### 2.7. Statistical Analysis

The experiment was set up as a completely randomized design and pots were randomly reallocated each week. Each analysis consisted of five replicates per treatment, unless otherwise stated. Two-way mixed effects analysis of variance (ANOVA) [47] was performed for all the data after verification of normality (Kolmogorov-Smirnov test) [47] and homogeneity of the variance (Hartley’s F_max_-test) [47]. The multiple comparisons of means were based on the minimum significant difference (MSD) method obtained from the *T* statistic for equal replicates or from the *T*’ statistic for not equal replicates in the case of cysteine, methionine and tryptophan analysis [47]. Pearson’s product-moment correlation coefficients and significance were calculated between Ce or Ti content in kernels and all the other measured parameters [47].

## 3. Results

### 3.1. Barley Biometric and Yield Parameters

Flag leaf area and 100-kernels weight were unaffected by the presence of cerium oxide (*n*CeO_2_) and titanium oxide (*n*TiO_2_) nanoparticles in the soil (Table 2). However, a reduction in flag leaf area and an increase in 100-kernels weight can be discerned under *n*CeO_2_ treatments but this is hidden by a high SE. Instead, nanoparticles treatments affected plants in terms of kernel quantity and grain yield, but the dose-responses are unclear for these traits (Table 2). In both *n*CeO_2_ and *n*TiO_2_ treatments the 500 mg·kg^−1^ level impaired kernel quantity and plant grain yield compared to the control; conversely, the 1000 mg·kg^−1^ treatments did not cause any significant limitation and, although not significant, there was a beneficial effect from the 1000 mg *n*TiO_2_·kg^−1^ treatment (Table 2).

### 3.2. Amylose and β-Glucans Concentrations in Kernels

The effects of *n*CeO_2_ and *n*TiO_2_ on amylose and β-glucans concentrations in barley kernels are displayed in Table 3. On the whole, metal nanoparticles (MeNPs) treatments had a marked effect on starch composition, as the amylose concentration significantly decreased (till 21% on average) compared to the control. However, there were no specific responses across the different MeNPs treatments. No significant differences were found in β-glucans concentration between control and MeNPs-treated plants.

### 3.3. Amino Acids Concentrations in Kernels

The effects of *n*CeO_2_ and *n*TiO_2_ in the soil on amino acid concentration and crude protein (CP) in kernels are displayed in Table 4. Overall, glutamic acid, proline and leucine were the most abundant amino acids in kernels with concentration ranges of 32–43, 15–21 and 9–12 mg·g^−1^ respectively (Table 4).

More in detail, *n*CeO_2_ and *n*TiO_2_ did not significantly modify concentrations of arginine and tryptophan but significantly and similarly increased cysteine, glutamic acid lysine and proline concentrations in all treatments with respect to the control. Aspartic acid concentration was unaffected at 500 mg·kg^−1^ of both *n*CeO_2_ and *n*TiO_2_ while significantly and similarly increased at 1000 mg·kg^−1^ of both *n*CeO_2_ and *n*TiO_2_.

The other amino acid responses varied depending on which MeNPs were added to the soil. In particular, *n*CeO_2_ treatments did not modify concentrations of alanine, glycine, histidine, isoleucine, leucine, phenylalanine, serine, tyrosine and valine. On the contrary, methionine and threonine concentrations significantly increased only at the highest *n*CeO_2_ treatment. Both *n*TiO_2_ treatments significantly increased alanine, glycine, isoleucine, leucine, methionine, threonine, tyrosine and valine concentrations. Conversely, only the highest *n*TiO_2_ treatment significantly increased histidine, phenylalanine and serine concentrations. However, in most amino acids the highest concentration was consistently measured at 1000 mg *n*TiO_2_·kg^−1^ treatment.

Finally, CP in kernels was significantly increased by approximately 19% in MeNPs-treated plants compared to the control (Table 4).

### 3.4. Ce and Ti Concentrations in Kernels

The effects of *n*CeO_2_ and *n*TiO_2_ in the soil on Ce and Ti concentrations in barley kernels are displayed in Table 5. We did not observe significant differences among the treatments for Ce and Ti concentrations in kernels as the SE of means were high (on average 45% of the mean). Nevertheless, a similar mean and a nearly homogeneous SE among treatments for Ce concentrations indicate that there were no differences among treatments. With regard to Ti, similar observations can be made except for the 1000 mg *n*TiO_2_·kg^−1^ treatment that had the highest mean but also a relatively high SE. Thus, although not significantly different, at the 1000 mg *n*TiO_2_·kg^−1^ treatment an enhancement of Ti can be envisaged with respect to the other treatments.

### 3.5. Nutrient Elements Concentrations in Kernels

Cerium oxide nanoparticles (*n*CeO_2_) treatments did not significantly modify several macro-nutrient concentrations (Ca, Mg, and P), Na (a beneficial nutrient) (Table 6) and all micronutrients except B (Table 7). Of the affected macro-nutrients, K was significantly lowered by both *n*CeO_2_ treatments, while S only by 1000 mg *n*CeO_2_·kg^−1^ treatment. Under 1000 mg *n*CeO_2_ treatment, B concentration was significantly lowered by approximately 62% compared to the control.

Regarding *n*TiO_2_ treatments, Ca was significantly increased and S significantly lowered by both *n*TiO_2_ treatments, while K was lowered only by 1000 mg *n*TiO_2_·kg^−1^ (Table 6). Among micronutrients, only Mn and Zn reached significantly higher concentrations at 500 mg *n*TiO_2_·kg^−1^ but not at 1000 mg *n*TiO_2_·kg^−1^ treatment (Table 7).

In all the treatments the Na/K ratio was unaffected while the Na/Ca ratio was significantly affected by both *n*TiO_2_ treatments (Table 6).

### 3.6. Pearson’s Product–Moment Correlation

Pearson’s product–moment correlation coefficients showed that only Mn (*r* = 0.859, *df* = 13, *p* < 0.001) concentration was positively and significantly correlated with Ce concentration in kernels from different concentrations of *n*CeO_2_ in soil. On the contrary, there were no significant correlations between Ti concentration in kernels and all the other measured parameters under *n*TiO_2_ treatments.

## 4. Discussion

### 4.1. Barley Biometric and Yield Parameters

There could be a parallel effect on source and sink organs in *n*CeO_2_ treated plants: the apparent reduction in flag leaf area is tentatively mirrored by a severe drop in sinks volume (kernel quantity). The severe reduction in kernel quantity under the 500 mg *n*CeO_2_·kg^−1^ treatment (and to a lesser extent under the 1000 mg *n*CeO_2_·kg^−1^ treatment), is associated with an increase in 100-kernel weight, but the compensation is not sufficient and the resulting plant grain yield was negatively affected. Our results are partially in agreement with those obtained by Rico *et al.* [16], who observed a reduced spike production in barley. Remarkably, these authors observed that plants exposed to 500 mg *n*CeO_2_·kg^−1^ did not form grains [16].

### 4.2. Amylose and β-Glucans in Kernels

Starch is the main carbohydrate in barley, accounting for approximately 65% to 75% of the grain dry weight. Amylose and amylopectin are the two components of starch. Barley types can be classified as normal (25%–27% amylose), waxy (below 5% amylose) and high-amylose (>35% amylose) [48]. The barley material used (cv. Tunika) was released as a two-row spring barley, and marketed in Italy for malt or feed production, as its high-amylose and moderate β-glucans traits are functional for both purposes. In general, the high-amylose trait in cereals is connected to resistant starch, which in turn has a positive role in human nutrition [49]. Amylose content has been tagged as the most sensitive parameter to heat stress in Japonica rice, maize and wheat [50], while there was no conclusive evidence for consistent changes in amylose content in barley grains exposed to high temperatures [51]. However, there was a decrease of amylose as a response to abiotic stress in our barley experiment.

Consistently with our results, Rico *et al.* [10] observed a lowered starch content in grain from rice treated with 500 mg *n*CeO_2_·kg^−1^ although significantly only in two of the three analyzed varieties. Conversely, Zhao *et al.* [14] did not observe significant differences in starch content in cucumber fruit. However, as well as analyzing a different species, they determined starch content in the whole cucumber fruit and not separated for skin, pulp and seeds [14].

Finally, as there were no significant differences in β-glucans concentration between control and MeNPs-treated plants, we hypothesize that MeNPs uptake has a limited impact on cell wall deposition in barley endosperm.

### 4.3. Amino Acids in Kernels

Our data on amino acid composition are consistent with other barley kernel composition studies [52,53]. In general, a total of 18 amino acids have been identified in barley proteins: with respect to animal growth requirements, lysine and threonine are the first and second most limiting amino acids, with methionine and tryptophan in third and fourth positions, respectively [53]. Since lysine is the first limiting amino acid in cereal grain protein, an increase in its level results in improved nutritional quality. However, high lysine strains and mutants (as Hiproly barley, found in the world barley collection in 1969) have been found to be associated with lower grain weight, and reduced yields.

A positive correlation between proline in leaves and plant stress is supported by a large body of data. Besides acting as an excellent osmolyte, proline plays different roles during stress, as a metal chelator, an antioxidative defense molecule and a signaling molecule [54]. An overproduction of proline, which in turn imparts stress tolerance by maintaining cell turgor or osmotic balance, is a common observation in stressful environments. We did not follow proline in leaf tissues, but MeNP-treated plants added on average 37% more proline in kernels compared to controls, which is the second largest increase, after the 51% increase in lysine. Metal nanoparticles (MeNP) treatments therefore probably operate as abiotic stressors on barley plants, and the observed proline increase in kernels could be related to the in-season proline evolution in green tissues. Contrasting results were reported by Rico *et al.* [13,16] for different amino acids in both barley and wheat exposed to *n*CeO_2_·kg^−1^.

Taking into account that MeNPs caused a decrease in grain yield per plant (−25%) comparable to the increase in CP (19%) compared to the control, the increased CP concentration could be due to less CP dilution by a reduced carbohydrate accumulation in MeNPs-treated plants.

From a different standpoint, the increased CP is in line with the increased total amino acids concentrations under MeNP treatments, on average 23% higher than in controls. Apart from tryptophan, similar effects were observed for each amino acid separately. Indeed, the magnitude of CP, total amino acids and single amino acid concentrations in MeNP treatments were comparable.

### 4.4. Ce and Ti in Kernels

As reported in our previous studies *in vitro* [21] and in kernels from shoots other than the main one [40], we did not observe significant differences among the treatments for Ce and Ti concentrations. This is in agreement with authors who studied different species under *n*CeO_2_ [13,15,19], or under *n*TiO_2_ exposure in soil [55]. It could be due to high data variation among replicates within treatments, but as the data overlapped a great deal among treatments, we are confident there is no enhanced Ce translocation into kernels under nanoparticle treatments compared to the control. An enhancement of Ti translocation under 1000 mg *n*TiO_2_·kg^−1^ treatment is instead notable. However, our results suggest that translocation of manufactured *n*CeO_2_ and *n*TiO_2_ (at least of nominal sizes of 25 nm) into barley kernels is hardly possible. Unfortunately, we were unable to test this hypothesis under TEM-EDAX analysis. In fact, due to the very hard seed coat, we were unable to cut kernels into slices, as required for TEM-EDAX analysis. Thus, additional efforts are needed to determine the content and distribution of Ce and Ti in the kernels. However, other authors observed that *n*CeO_2_ treatments largely increased Ce concentration in fruits and seeds of different species [10,36,56] including barley [16]. The contrasting data reported in barley by Rico *et al.* [16] can be explained by the different physical-chemical properties of the nanoparticles used in the two studies as discussed in more detail below in Section 4.5. A root to fruit translocation of *n*TiO_2_ was also observed in cucumber after plant exposure to *n*TiO_2_ in soil [57].

### 4.5. Nutrient Elements in Kernels

Potassium is the most abundant cation in cytoplasm, actively maintaining osmotic potential and stabilizing pH between 7 and 8, the optimum range for most enzyme activities. As it is also necessary for protein synthesis and other metabolic processes [6], its reduction at both *n*CeO_2_ levels and at 1000 mg *n*TiO_2_·kg^−1^ treatment could have a negative impact on enzyme activities (and kernel quality).

Sulfur is a structural constituent of coenzymes and secondary plant products containing amino acids cysteine and methionine and can also act as a functional group directly involved in metabolic reactions [6]. Therefore the reduction in S that occurred at both *n*TiO_2_ treatments could theoretically affect glutathione synthesis and the antioxidant capacity of kernels. However, the expected reduction in cysteine and methionine was not observed; in fact, they were enhanced (Table 4). We speculate that even if S translocation to the kernels was lowered, the small amount of available S was nonetheless effectively directed to protein synthesis, avoiding its use for other purposes.

Boron is involved in a number of metabolic pathways and the severe reduction in B concentration could indicate a restricted permeation of B due to *n*CeO_2_ presence.

Cerium oxide nanoparticles interfered with Mn, demonstrating a tendency for Mn accumulation at increasing levels of Ce in kernels as demonstrated by a significant Pearson’s product-moment correlation. With respect to the few published articles about Ce correlation with other elements in grains of barley and other species, we did not find any evidence of correlation between other elements and Ce [10,13,16].

Other authors reported contrasting data and data only partially in agreement with our results. For example, similarly to our results, Rico *et al.* [10] observed no changes in concentrations of most of the nutrients in grain from three rice varieties grown in soil with 500 mg *n*CeO_2_·kg^−1^ compared to the control. Notable exceptions regarded Al, Fe, K, Na and S, which responded differently in the three analyzed varieties, indicating not only a dose effect but also a variety response. Also, wheat grown in soil exposed to *n*CeO_2_ concentrations lower than 500 mg·kg^−1^ showed no change in kernel nutrients except for S and Mn which had an inverse hormetic response [13].

Rico *et al.* [16] observed significant increases in both macro- and micro-nutrients in grains from barley plants exposed to *n*CeO_2_ at different levels, which is in disagreement with our observations. Peralta-Videa *et al.* [12] reported no effects on S and K content in pods from soybean plants grown at *n*CeO_2_ treatments similar to ours. Aluminum, Na and Ca concentrations in soybean pods were negatively affected, while Cu and P were increased at 1000 mg *n*CeO_2_·kg^−1^ treatment [12]. Different results were also obtained by Zhao *et al.* [14], who analyzed mineral content of cucumber fruit from plants grown at comparable *n*CeO_2_ concentrations to ours. These authors did not observe significant differences in any element concentration between treated and control plants; the exceptions were Mo that decreased at both treatments and Mg that had a hormetic response [14]. Finally, under *n*CeO_2_ treatments comparable to ours, significant increases of K and Mn were observed in undeveloped corn cobs but not in developed ones [15].

Contrary to *n*CeO_2_, scant results are available about the effects of plants grown in soil containing *n*TiO_2_. Only Servin *et al.* [57] reported data from a full mineral analysis of cucumber fruit. They added *n*TiO_2_ to soil at concentrations from 250 to 750 mg *n*TiO_2_·kg^−1^ [57]. There was no substantial effect on macro- and micro-nutrients in fruits, apart from P and K, in which a hormetic effect was detected [57]. Instead, and in agreement with our results, Klingenfuss [55] reported no significant differences in P concentrations in wheat grains after exposure of plants in soil with a range of *n*TiO_2_ varying from 1 to 1000 mg *n*TiO_2_·kg^−1^. Unfortunately, Klingenfuss did not report the concentrations of other nutrients [55]. However, these results can indicate a species-specific response and possibly diverse effects on mineral concentrations in eudicots and monocots, with the latter being more affected.

The high levels of Ca in both kernels and vegetative tissues such as roots and shoots [40] could be linked to the higher yield grain under *n*TiO_2_ treatments. Calcium is a messenger between environmental factors and plant responses in terms of growth and development [6]. *n*TiO_2_ (or the released ionic form) could potentially have a beneficial effect enhancing absorption and translocation of Ca [58]. Moreover, other authors showed that seeds immersed in *n*TiO_2_ solution and sprayed with *n*TiO_2_ over shoots could enhance plant growth through several mechanisms including the enhancement of photosynthesis, enzyme activity and even by a supposed new N_2_ fixation mechanism in the air [32,33,34,35,59]. However, in our experiment we included *n*TiO_2_ in the soil and the observed benefits could be explained by translocation of at least minimum amounts of *n*TiO_2_ in shoots.

The Na/K and Na/Ca ratios are useful indicators of plant response to stress and can also indicate kernels quality. The unaffected Na/K ratio indicates no major metabolic disorders [60]. On the contrary the Na/Ca ratio was reduced by 2.4-fold on average in both *n*TiO_2_ treatments, which suggests an increased competitive inhibition between absorption of Na and Ca that can possibly mitigate eventually harmful effects of Ti—similarly to what was observed for rice under *n*CeO_2_ exposure [10]. Alternatively, as Na concentrations did not change significantly from the control whereas Ca concentrations increased significantly under *n*TiO_2_ treatments, this could be due to a beneficial effect of *n*TiO_2_ that can enhance Ca absorption and translocation as observed above. Curiously, this is not the case for *n*CeO_2,_ although other authors reported a reduction in the Na/Ca ratio for rice grains after exposure of plants to *n*CeO_2_ in soil [10]. In kernels under *n*TiO_2_ treatments, the reduction in Na/Ca ratio and increased concentration of Ca can be beneficial for human nutrition on the one hand, but can negatively affect the eating quality of kernels on the other.

A separate comment needs to be made about the contrasting data reported in the only barley full life cycle study performed [16]. Besides the fact that the authors cultivated another variety of barley in a different soil, the contrasting results obtained from our experiments and also supported by the low Ce quantity found in kernels from shoots other than the main one [40] can tentatively be explained by different *n*CeO_2_ sizes (8 nm *vs.* 22.7 nm) and the methodology used to apply MeNPs to the soil. In fact, Rico *et al.* [16] sonicated a *n*CeO_2_ solution prior to diluting it in soil and mixing, thus possibly making *n*CeO_2_ more bioavailable. It is also possible that a larger quantity of ionic Ce, toxic to plants [28,30], was released in soil by *n*CeO_2_ in the experiment by Rico *et al.* [16] which is supported by their larger BET surface area (93.8 *vs.* 46.1 m^2^·g^−1^).

## 5. Conclusions

Among the metal nanoparticle (MeNP) treatments, some significant effects (on kernel numbers, 100-kernel weight, and Zn and Mn concentrations) were visible only at lower concentrations and not at higher concentrations (hormetic response). Different nanoparticles can therefore positively or negatively influence different nutritional values. Although the observed differences between 500 and 1000 mg·kg^−1^ MeNP treatments might be due to MeNP interactions (agglomeration and/or association) with soil constituents, this hypothesis remains to be tested at higher concentrations. Indeed, toxicity of MeNPs could be closely related to their chemical composition, structure, particle size and surface area. Substantial research is needed on MeNP size, their physico-chemical characteristics and their interactions with soil components.

Both MeNPs had a negative impact on the amylose content of kernels, with a reduction in grain yield. This was associated with an increase in crude protein (CP) and most amino acids. Interestingly, lysine, the essential and largely deficient amino acid in cereal grains, showed the largest percentage increase.

In terms of MeNPs impact on barley food production, our data are far from being exhaustive; however, a possible negative impact on kernel energy content, K and S concentrations might be counterbalanced by CP and lysine increase. Moreover, *n*TiO_2_ treatments also increased Ca, Mg and Zn kernel concentrations.

Our findings demonstrate that cerium oxide nanoparticles (*n*CeO_2_) and *n*TiO_2_ act differently on the nutritional quality of barley kernels. Generally, barley kernels were found to be more negatively affected by *n*CeO_2_, while *n*TiO_2_ can potentially have a beneficial effect. This study provides the first proof that *n*CeO_2_ and *n*TiO_2_ nanoparticles can have significant and contrasting impacts on the composition and nutritional value of barley kernels.

## Figures and Tables

**Table 1:** Characteristics of soil used in this study (*n* = 5).

Parameter	Mean ± SE
Clay (%)	26.0 ± 0.0
Silt (%)	6.4 ± 0.4
Sand (%)	67.6 ± 0.4
pH	7.44 ± 0.01
EC (μS·cm^−1^) at 25 °C	1235 ± 194
CEC (cmol·kg^−1^ dw)	13.87 ± 0.30
Available P (μg·g^−1^ dw)	61.3 ± 8.4
Total carbonate (g·kg^−1^)	72.0 ± 14.3
Total organic C (%)	2.22 ± 0.27
Total N (%)	0.17 ± 0.01

**Table 2:** Biometric and yield parameters of barley plants in soil treated with none (Control), 500 mg *n*CeO_2_·kg^−1^, 1000 mg *n*CeO_2_·kg^−1^, 500 mg *n*TiO_2_·kg^−1^ and 1000 mg *n*TiO_2_·kg^−1^.

Treatment	Flag Leaf Area (cm^2^)	Kernels (n·plant^−1^)	Grain Yield (g·plant^−1^)	100-Kernels Weight (g)
Control	13.36 ± 1.10 a ^1^	117.0 ± 13.2 a	4.4 ± 0.6 a	3.76 ± 0.19 ab
500 mg *n*CeO_2_·kg^−1^	10.75 ± 2.21 a	53.2 ± 6.0 b	2.4 ± 0.3 b	4.60 ± 0.08 a
1000 mg *n*CeO_2_·kg^−1^	10.55 ± 2.98 a	90.6 ± 7.5 ab	3.7 ± 0.3 ab	4.13 ± 0.31 ab
500 mg *n*TiO_2_·kg^−1^	15.43 ± 2.79 a	66.2 ± 10.6 b	2.1 ± 0.4 b	3.04 ± 0.26 b
1000 mg *n*TiO_2_·kg^−1^	14.31 ± 1.75 a	129.8 ± 18.3 a	5.1 ± 0.6 a	4.04 ± 0.37 ab

^1^ Values are means ± SE (*n* = 5). Different letters indicate significant differences between treatments (*p* ≤ 0.05, *T* test) for each yield parameter separately.

**Table 3:** β-Glucans and amylose concentration in barley kernels at ripening from main shoot grown in soil treated with none (Control), 500 mg *n*CeO_2_·kg^−1^, 1000 mg *n*CeO_2_·kg^−1^, 500 mg *n*TiO_2_·kg^−1^ and 1000 mg *n*TiO_2_·kg^−1^.

Treatment	β-Glucans (% dw)	Amylose (% dw)
Control	5.10 ± 0.19 a ^1^	52.14 ± 1.34 a
500 mg *n*CeO_2_·kg^−1^	4.80 ± 0.19 a	43.85 ± 2.10 b
1000 mg *n*CeO_2_·kg^−1^	4.52 ± 0.11 a	39.74 ± 1.19 b
500 mg *n*TiO_2_·kg^−1^	4.54 ± 0.13 a	39.59 ± 2.08 b
1000 mg *n*TiO_2_·kg^−1^	4.94 ± 0.17 a	41.49 ± 1.65 b

^1^ Values are means ± SE (*n* = 5). Different letters indicate significant differences between treatments (*p* ≤ 0.05, *T* test) for each element separately.

**Table 4:** Amino acid (mg·g^−1^) and crude protein (CP, %) concentration in barley kernels at ripening from main shoot grown in soil treated with none (Control), 500 mg *n*CeO_2_·kg^−1^, 1000 mg *n*CeO_2_·kg^−1^, 500 mg *n*TiO_2_·kg^−1^ and 1000 mg *n*TiO_2_·kg^−1^.

Parameter	Control	500 mg *n*CeO_2_·kg^−1^	1000 mg *n*CeO_2_·kg^−1^	500 mg *n*TiO_2_·kg^−1^	1000 mg *n*TiO_2_·kg^−1^
Alanine	5.65 ± 0.23 c ^1^	6.06 ± 0.12 bc	6.71 ± 0.31 ac	7.35 ± 0.47 a	6.75 ± 0.07 ab
Arginine	7.55 ± 0.59 a	8.72 ± 0.33 a	8.51 ± 0.32 a	9.26 ± 0.25 a	9.12 ± 0.37 a
Aspartic acid	7.18 ± 0.30 b	8.28 ± 0.35 ab	8.50 ± 0.37 a	8.58 ± 0.29 ab	9.09 ± 0.22 a
Cysteine ^2^	6.85 ± 0.06 b	8.70 ± 0.17 a	8.04 ± 0.15 a	8.07 ± 0.00 a	8.42 ± 0.16 a
Glutamic acid	31.98 ± 1.60 b	38.97 ± 2.10 a	39.79 ± 1.08 a	40.75 ± 1.67 a	43.05 ± 0.82 a
Glycine	5.98 ± 0.20 c	6.07 ± 0.24 c	6.79 ± 0.29 bc	7.74 ± 0.13 ab	8.01 ± 0.19 a
Histidine	3.14 ± 0.23 b	3.54 ± 0.19 ab	3.46 ± 0.15 ab	3.66 ± 0.08 ab	3.89 ± 0.08 a
Isoleucine	5.29 ± 0.21 b	6.03 ± 0.29 ab	6.02 ± 0.20 ab	6.42 ± 0.16 a	6.77 ± 0.11 a
Leucine	9.40 ± 0.33 b	10.78 ± 0.52 ab	10.81 ± 0.31 ab	11.24 ± 0.36 a	11.70 ± 0.19 a
Lysine	3.67 ± 0.14 c	5.02 ± 0.23 b	5.34 ± 0.21 ab	5.85 ± 0.15 ab	5.98 ± 0.20 a
Methionine	2.39 ± 0.06 b	2.72 ± 0.11 ab	2.87 ± 0.09 a	3.08 ± 0.00 a	3.00 ± 0.09 a
Phenylalanine	7.48 ± 0.42 b	8.68 ± 0.53 ab	8.66 ± 0.22 ab	9.12 ± 0.29 ab	9.37 ± 0.20 a
Proline	14.85 ± 0.75 b	19.23 ± 1.29 a	20.16 ± 0.55 a	20.44 ± 1.36 a	21.44 ± 0.63 a
Serine	5.84 ± 0.24 b	6.57 ± 0.38 ab	6.71 ± 0.16 ab	6.78 ± 0.16 ab	6.84 ± 0.08 a
Threonine	4.31 ± 0.14 c	4.70 ± 0.22 bc	5.12 ± 0.14 ab	5.11 ± 0.16 ab	5.35 ± 0.04 a
Tryptophan	1.15 ± 0.30 a	0.89 ± 0.19 a	0.94 ± 0.26 a	0.53 ± 0.00 a	0.74 ± 0.08 a
Tyrosine	3.36 ± 0.19 b	3.39 ± 0.18 b	3.78 ± 0.16 ab	4.34 ± 0.09 a	4.22 ± 0.16 a
Valine	7.04 ± 0.22 b	7.80 ± 0.35 ab	7.80 ± 0.24 ab	8.29 ± 0.29 a	8.68 ± 0.18 a
Total	133.22 ± 5.01 b	155.98 ± 6.81 a	160.17 ± 4.01 a	166.58 ± 4.77 a	172.46 ± 2.36 a
CP	12.08 ± 0.45 b	13.34 ± 0.65 ab	14.55 ± 0.30 a	14.30 ± 0.64 a	15.17 ± 0.30 a

^1^ Values are means ± SE (*n* = 5 except for cysteine, methionine and tryptophan were *n* = 2 or 3). Different letters indicate significant differences between treatments (*p* ≤ 0.05, *T* or *T*’ test) for each amino acid separately; ^2^ Cysteine is expressed as cysteic acid.

**Table 5:** Cerium and titanium concentration in barley kernels at ripening from main shoot grown in soil treated with none (Control), 500 mg *n*CeO_2_·kg^−1^, 1000 mg *n*CeO_2_·kg^−1^, 500 mg *n*TiO_2_·kg^−1^ and 1000 mg *n*TiO_2_·kg^−1^.

Treatment	Ce (mg·kg^−1^ dw)	Ti (mg·kg^−1^ dw)
Control	0.50 ± 0.20 a ^1^	2.19 ± 1.19 a
500 mg *n*CeO_2_·kg^−1^	1.03 ± 0.84 a	1.27 ± 0.29 a
1000 mg *n*CeO_2_·kg^−1^	0.70 ± 0.37 a	0.85 ± 0.12 a
500 mg *n*TiO_2_·kg^−1^	1.08 ± 0.54 a	1.70 ± 0.53 a
1000 mg *n*TiO_2_·kg^−1^	1.16 ± 0.61 a	8.72 ± 4.76 a

^1^ Values are means ± SE (*n* = 5). Different letters indicate significant differences between treatments (*p* ≤ 0.05, *T* test) for each element separately.

**Table 6:** Concentration of macro-nutrients, Na, and Na/K and Na/Ca ratios in barley kernels at ripening from main shoot grown in soil treated with none (Control), 500 mg *n*CeO_2_·kg^−1^, 1000 mg *n*CeO_2_·kg^−1^, 500 mg *n*TiO_2_·kg^−1^ and 1000 mg *n*TiO_2_·kg^−1^.

Treatment	Ca (mg·kg^−1^ dw)	K (mg·kg^−1^ dw)	Mg (mg·kg^−1^ dw)	Na (mg·kg^−1^ dw)	P (mg·kg^−1^ dw)	S (mg·kg^−1^ dw)	Na/K	Na/Ca
Control	377 ± 21 c ^1^	4570 ± 105 a	1881 ± 59 a	187 ± 22 a	4549 ± 335 a	4758 ± 199 a	0.04 ± 0.01 a	0.50 ± 0.05 a
500 mg *n*CeO_2_·kg^−1^	387 ± 11 bc	3792 ± 82 b	1756 ± 22 a	150 ± 31 a	4519 ± 110 a	4162 ± 266 ab	0.04 ± 0.01 a	0.38 ± 0.07 ab
1000 mg *n*CeO_2_·kg^−1^	426 ± 19 bc	3755 ± 75 b	1765 ± 57 a	133 ± 18 a	4511 ± 312 a	3620 ± 144 bc	0.04 ± 0.01 a	0.32 ± 0.05 ab
500 mg *n*TiO_2_·kg^−1^	661 ± 81 a	4187 ± 157 ab	1983 ± 124 a	152 ± 17 a	4867 ± 234 a	3148 ± 80 c	0.04 ± 0.01 a	0.24 ± 0.04 b
1000 mg *n*TiO_2_·kg^−1^	543 ± 8 ab	3762 ± 77 b	1736 ± 39 a	95 ± 26 a	4359 ± 314 a	3194 ± 220 c	0.03 ± 0.01 a	0.18 ± 0.05 b

^1^ Values are means ± SE (*n* = 5). Different letters indicate significant differences between treatments (*p* ≤ 0.05, *T* test) for each element separately.

**Table 7:** Concentration of micro-nutrients in barley kernels at ripening from main shoot grown in soil treated with none (control), 500 mg *n*CeO_2_·kg^−1^, 1000 mg *n*CeO_2_·kg^−1^, 500 mg *n*TiO_2_·kg^−1^ and 1000 mg *n*TiO_2_·kg^−1^.

Treatment	B (mg·kg^−1^ dw)	Cu (mg·kg^−1^ dw)	Fe (mg·kg^−1^ dw)	Mn (mg·kg^−1^ dw)	Ni (mg·kg^−1^ dw)	Zn (mg·kg^−1^ dw)
Control	8.64 ± 1.02 ab ^1^	8.91 ± 1.33 a	38.81 ± 6.51 a	18.80 ± 0.64 b	0.44 ± 0.14 a	55.74 ± 5.36 b
500 mg *n*CeO_2_·kg^−1^	15.12 ± 4.73 a	8.01 ± 0.80 a	37.52 ± 2.61 a	21.87 ± 1.17 ab	0.34 ± 0.07 a	54.07 ± 2.55 b
1000 mg *n*CeO_2_·kg^−1^	3.32 ± 1.37 b	6.99 ± 0.31 a	28.49 ± 1.67 a	19.84 ± 0.68 b	0.39 ± 0.04 a	56.69 ± 1.18 b
500 mg *n*TiO_2_·kg^−1^	8.01 ± 1.70 ab	7.52 ± 1.16 a	34.25 ± 2.17 a	25.10 ± 1.06 a	0.75 ± 0.17 a	69.63 ± 2.61 a
1000 mg *n*TiO_2_·kg^−1^	6.02 ± 1.58 ab	8.24 ± 0.28 a	38.35 ± 4.47 a	21.59 ± 1.23 ab	0.32 ± 0.08 a	59.59 ± 1.34 ab

^1^ Values are means ± SE (*n* = 5). Different letters indicate significant differences between treatments (*p* ≤ 0.05, *T* test) for each element separately.

## References

[1] Roco M.C., Roco M.C., Mirkin C.A., Hersam M.C. (2010). The long view of nanotechnology development: The national nanotechnology initiative at 10 years. Nanotechnology Research Directions for Societal Needs in 2020: Retrospective and Outlook.

[2] Nadeau S., Bouchard M., Debia M., DeMarcellis-Warin N., Hallé S., Songmene V., Therrien M.-C., Wilkinson K., Ateme-Nguema B., Dufour G. (2013). ÉquiNanos: Innovative team for nanoparticle risk management. Nanomed. Nanotechnol. Biol. Med..

[3] International Organization for Standardization (2010). Nanotechnologies-Vocabulary-Part 1: Core Terms.

[4] Gruère G.P. (2012). Implications of nanotechnology growth in food and agriculture in OECD countries. Food Policy.

[5] Takeuchi M.T., Kojima M., Luetzow M. (2014). State of the art on the initiatives and activities relevant to risk assessment and risk management of nanotechnologies in the food and agriculture sectors. Food Res. Int..

[6] Marschner H. (1995). Mineral Nutrition of Higher Plants.

[7] Zhu H., Han J., Xiao J.Q., Jin Y. (2008). Uptake, translocation, and accumulation of manufactured iron oxide nanoparticles by pumpkin plants. J. Environ. Monit..

[8] Miralles P., Church T.L., Harris A.T. (2012). Toxicity, uptake, and translocation of engineered nanomaterials in vascular plants. Environ. Sci. Technol..

[9] Zhao L.J., Peralta-Videa J.R., Hernandez-Viezcas J.A., Hong J., Gardea-Torresdey J.L. (2012). Transport and retention behavior of ZnO nanoparticles in two natural soils: Effect of surface coating and soil composition. J. Nano Res..

[10] Rico C.M., Morales M.I., Barrios A.C., McCreary R., Hong J., Lee W.Y., Nunez J., Peralta-Videa J.R., Gardea-Torresdey J.L. (2013). Effect of cerium oxide nanoparticles on the quality of rice (*Oryza sativa* L.) grains. J. Agric. Food. Chem..

[11] Schwabe F., Schulin R., Limbach L.K., Stark W., Bürge D., Nowack B. (2013). Influence of two types of organic matter on interaction of CeO_2_ nanoparticles with plants in hydroponic culture. Chemosphere.

[12] Peralta-Videa J.R., Hernandez-Viezcas J.A., Zhao L., Diaz B.C., Ge Y., Priester J.H., Holden P.A., Gardea-Torresdey J.L. (2014). Cerium dioxide and zinc oxide nanoparticles alter the nutritional value of soil cultivated soybean plants. Plant Physiol. Biochem..

[13] Rico C.M., Lee S.C., Rubenecia R., Mukherjee A., Hong J., Peralta-Videa J.R., Gardea-Torresdey J.L. (2014). Cerium oxide nanoparticles impact yield and modify nutritional parameters in wheat (*Triticum aestivum* L.). J. Agric. Food Chem..

[14] Zhao L., Peralta-Videa J.R., Rico C.M., Hernandez-Viezcas J.A., Sun Y., Niu G., Servin A., Nunez J.E., Duarte-Gardea M., Gardea-Torresdey J.L. (2014). CeO_2_ and ZnO nanoparticles change the nutritional qualities of cucumber (*Cucumis sativus*). J. Agric. Food Chem..

[15] Zhao L., Sun Y., Hernandez-Viezcas J.A., Hong J., Majumdar S., Niu G., Duarte-Gardea M., Peralta-Videa J.R., Gardea-Torresdey J.L. (2015). Monitoring the environmental effects of CeO_2_ and ZnO nanoparticles through the life cycle of corn (*Zea mays*) plants and *in situ* μ-XRF mapping of nutrients in kernels. Environ. Sci. Technol..

[16] Rico C.M., Barrios A.C., Tan W., Rubenecia R., Lee S.C., Varela-Ramirez A., Peralta-Videa J.R., Gardea-Torresdey J.L. (2015). Physiological and biochemical response of soil-grown barley (*Hordeum vulgare* L.) to cerium oxide nanoparticles. Environ. Sci. Pollut. Res. Int..

[17] Organization for Economic Cooperation and Development List of Manufactured Nanomaterials and List of Endpoints for Phase One of the Sponsorship Programme for the Testing on Manufactured Nanomaterials: Revision. http://www.oecd.org/officialdocuments/publicdisplaydocumentpdf/?cote=env/jm/mono(2010)46&doclanguage=en.

[18] López-Moreno M.L., de la Rosa G., Hernández-Viezcas J.A., Castillo-Michel H., Botez C.E., Peralta-Videa J.R., Gardea-Torresdey J.L. (2010). Evidence of the differential biotransformation and genotoxicity of ZnO and CeO_2_ nanoparticles on soybean (*Glycine max*) plants. Environ. Sci. Technol..

[19] López-Moreno M.L., de La Rosa G., Hernández-Viezcas J.A., Peralta-Videa J.R., Gardea-Torresdey J.L. (2010). X-ray Absorption Spectroscopy (XAS) corroboration of the uptake and storage of CeO_2_ nanoparticles and assessment of their differential toxicity in four edible plant species. J. Agric. Food Chem..

[20] Gomez-Garay A., Pintos B., Manzanera J.A., Lobo C., Villalobos N., Martín L. (2014). Uptake of CeO_2_ nanoparticles and its effect on growth of *Medicago arborea in vitro* plantlets. Biol. Trace Elem. Res..

[21] Mattiello A., Filippi A., Pošćić F., Musetti R., Salvatici M.C., Giordano C., Vischi M., Bertolini A., Marchiol L. (2015). Evidence of phytotoxicity and genotoxicity in *Hordeum vulgare* L. exposed to CeO_2_ and TiO_2_ nanoparticles. Front. Plant Sci..

[22] Hong F., Zhou J., Liu C., Yang F., Wu C., Zheng L., Yang P. (2005). Effect of nano-TiO_2_ on photochemical reaction of chloroplasts of spinach. Biol. Trace Elem. Res..

[23] Zheng L., Hong F., Lu S., Liu C. (2005). Effect of nano-TiO_2_ on strength of naturally aged seeds and growth of spinach. Biol. Trace Elem. Res..

[24] Asli S., Neumann M. (2009). Colloidal suspensions of clay or titanium dioxide nanoparticles can inhibit leaf growth and transpiration via physical effects on root water transport. Plant Cell Environ..

[25] Zhang Z., He X., Zhang H., Ma Y., Zhang P., Ding Y., Zhao Y. (2011). Uptake and distribution of ceria nanoparticles in cucumber plants. Metallomics.

[26] Larue C., Laurette J., Herlin-Boime N., Khodja H., Fayard B., Flank A.M., Brisset F., Carriere M. (2012). Accumulation, translocation and impact of TiO_2_ nanoparticles in wheat (*Triticum aestivum* spp.): Influence of diameter and crystal phase. Sci. Total Environ..

[27] Jacob D.L., Borchardt J.D., Navaratnam L., Otte M.L., Bezbaruah A.N. (2013). Uptake and translocation of Ti from nanoparticles in crops and wetland plants. Int. J. Phytoremediat..

[28] Zhang W., Ebbs S.D., Musante C., White J.C., Gao C., Ma X. (2015). Uptake and accumulation of bulk and nanosized cerium oxide particles and ionic cerium by radish (*Raphanus sativus* L.). J. Agric. Food Chem..

[29] Dehkourdi E.H., Mosavi M. (2013). Effect of anatase nanoparticles (TiO_2_) on parsley seed germination (*Petroselinum crispum*) *in vitro*. Biol. Trace Elem. Res..

[30] Cui D., Zhang P., Ma Y., He X., Li Y., Zhang J., Zhao Y., Zhang Z. (2014). Effect of cerium oxide nanoparticles on asparagus lettuce cultured in an agar medium. Environ. Sci. Nano.

[31] Hong F., Yang F., Liu C., Gao Q., Won Z., Gu F., Wu C., Ma Z., Zhou J., Yang P. (2005). Influences of nano-TiO_2_ on the chloroplast aging of spinach under light. Biol. Trace Elem. Res..

[32] Gao F., Hong F., Liu C., Zheng L., Su M., Wu X., Yang F., Wu C., Yang P. (2006). Mechanism of nano-anatase TiO_2_ on promoting photosynthetic carbon reaction of spinach. Biol. Trace Elem. Res..

[33] Yang F., Liu C., Gao F., Su M., Wu X., Zheng L., Hong F., Yang P. (2007). The improvement of spinach growth by nano-anatase TiO_2_ treatment is related to nitrogen photoreduction. Biol. Trace Elem. Res..

[34] Gao F., Liu C., Qu C., Zheng L., Yang F., Su M., Hong F. (2008). Was improvement of spinach growth by nano-TiO_2_ treatment related to the changes of Rubisco activase?. Biometals.

[35] Ling M., Chao L., Chun Q., Si Y., Jie L., Feng G., Fa H. (2008). Rubisco activase mRNA expression in spinach: Modulation by nanoanatase treatment. Biol. Trace Elem. Res..

[36] Wang Q., Ma X., Zhang W., Pei H., Chen Y. (2012). The impact of cerium oxide nanoparticles on tomato (*Solanum lycopersicum* L.) and its implications for food safety. Metallomics.

[37] Food and Agriculture Organization of the United Nations FAOSTAT. http://faostat3.fao.org/download/Q/QC/E.

[38] Delogu G., Cattivelli L., Pecchioni N., De Falcis D., Maggiore T., Stanca A.M. (1998). Uptake and agronomic efficiency of nitrogen in winter barley and winter wheat. Eur. J. Agron..

[39] Pansu M., Gautheyrou J. (2006). Handbook of Soil Analysis: Mineralogical, Organic and Inorganic Methods.

[40] Marchiol L., Mattiello A., Pošćić F., Fellet G., Zavalloni C., Carlino E., Musetti R. (2016). Changes in physiological and agronomical parameters of barley (*Hordeum vulgare*) exposed to cerium and titanium dioxide nanoparticles. Int. J. Environ. Res. Public Health.

[41] Sun T.Y., Bornhöft N.A., Hungerbühler K., Nowack B. (2016). Dynamic probabilistic modeling of environmental emissions of engineered nanomaterials. Environ. Sci. Technol..

[42] Du W., Tan W., Peralta-Videa J.R., Gardea-Torresdey J.L., Ji R., Yin Y., Guo H. (2016). Interaction of metal oxide nanoparticles with higher terrestrial plants: Physiological and biochemical aspects. Plant Physiol. Biochem..

[43] Zadoks J.C., Chang T.T., Konzak C.F. (1974). A decimal code for the growth stages of cereals. Weed Res..

[44] Bosch L., Alegria A., Farrè R. (2006). Application of the 6-aminoquinolyl-*N*-hydroxysuccinimidyl carbamate (AQC) reagent to the RP-HPLC determination of amino acids in infant foods. J. Chromatogr. B.

[45] Liu H.J., Chang B.Y., Yan H.W., Yu F.H., Lu X.X. (1995). Determination of amino acids in food and feed by derivatization with 6-aminoquinolyl-*N*-hydroxysuccinimidyl carbamate and reversed-phase liquid chromatographic separation. J. AOAC Int..

[46] Mariotti F., Tomé D., Mirand P.P. (2008). Converting nitrogen into protein-beyond 6.25 and Jones’ factors. Crit. Rev. Food Sci. Nutr..

[47] Sokal R.R., Rohlf F.J. (2010). Biometry. The Principles and Practice of Statistics in Biological Research.

[48] Shu X., Rasmussen S.K. (2014). Quantification of amylose, amylopectin, and β-glucan in search for genes controlling the three major quality traits in barley by genome-wide association studies. Front. Plant Sci..

[49] Berry C.S. (1986). Resistant starch: Formation and measurement of starch that survives exhaustive digestion with amylolytic enzymes during the determination of dietary fibre. J. Cereal Sci..

[50] Beckles D.B., Thitisaksul M. (2014). How environmental stress affects starch composition and functionality in cereal endosperm. Starch-Stärke.

[51] Kiseleva V.I., Tester R.F., Wasserman L.A., Krivandin A.V., Popov A.A., Yuryev V.P. (2013). Influence of growth temperature on the structure and thermodynamic parameters of barley starches. Carbohyd. Polym..

[52] Pomeranz Y., Robbins G.S., Smith R.T., Craddock J.C., Gilbertson J.T. (1976). Protein content and amino acid composition of barleys from the world collection. Cereal Chem..

[53] Newman R.K., Newman C.W. (2008). Barley for Food and Health: Science, Technology and Products.

[54] Hayat S., Hayat Q., Alyemeni M.N., Wani A.S., Pichtel J., Ahmad A. (2012). Role of proline under changing environments. Plant. Signal. Behav..

[55] Klingenfuss F. (2014). Testing of TiO_2_ Nanoparticles on Wheat and Microorganisms in a Soil Microcosm. Master’s Thesis.

[56] Priester J.H., Ge Y., Mielke R.E., Horst A.M., Cole Moritz S., Espinosa K., Gelb J., Walker S.L., Nisbet R.M., An Y.-J. (2012). Soybean susceptibility to manufactured nanomaterials with evidence for food quality and soil fertility interruption. Proc. Natl. Acad. Sci. USA.

[57] Servin A.D., Morales M.I., Castillo-Michel H., Hernandez-Viezcas J.A., Munoz B., Zhao L., Nunez J.E., Peralta-Videa J.R., Gardea-Torresdey J.L. (2013). Synchrotron verification of TiO_2_ accumulation in cucumber fruit: A possible pathway of TiO_2_ nanoparticle transfer from soil into the food chain. Environ. Sci. Technol..

[58] Alcaraz-Lopez C., Botia M., Alcaraz C.F., Riquelme F. (2003). Effects of foliar sprays containing calcium, magnesium and titanium on plum (*Prunus domestica* L.) fruit quality. J. Plant Physiol..

[59] Su M., Liu H., Liu C., Qu C., Zheng L., Hong F. (2009). Promotion of nano-anatase TiO_2_ on the spectral responses and photochemical activities of D1/D2/Cyt b559 complex of spinach. Spectrochim. Acta Part A Mol. Biomol. Spectrosc..

[60] Brady C.J., Gibson T.S., Barlow E.W.R., Speirs J., Wyn Jones R.G. (1984). Salt-tolerance in plants. I. Ions, compatible organic solutes and the stability of plant ribosomes. Plant Cell Environ..

